# The meaning of labour pain: how the social environment and other contextual factors shape women’s experiences

**DOI:** 10.1186/s12884-017-1343-3

**Published:** 2017-05-30

**Authors:** Laura Y. Whitburn, Lester E. Jones, Mary-Ann Davey, Rhonda Small

**Affiliations:** 1 School of Life Sciences & Judith Lumley Centre, La Trobe University, Bundoora, Victoria 3086 Australia; 2 Judith Lumley Centre, La Trobe University, Bundoora, Victoria 3086 Australia; 3 Department of Obstetrics and Gynaecology, Monash University, Clayton, Victoria 3800 Australia

**Keywords:** Labour pain, Childbirth, Social support, Pain cognitions, Pain control, Pain science, Women’s health, Phenomenology

## Abstract

**Background:**

The majority of women experience pain during labour and childbirth, however not all women experience it in the same way. In order to develop a more complete understanding of labour pain, this study aimed to examine women’s experiences within the perspective of modern pain science. A more complete understanding of this phenomenon can then guide the development of interventions to enhance women’s experiences and potentially reduce their need for pharmacological intervention.

**Methods:**

A qualitative study was conducted using phenomenology as the theoretical framework. Data were collected from 21 nulliparous women, birthing at one of two large maternity services, through face-to-face interviews and written questionnaires. Data were analysed using an Interpretative Phenomenological Analysis approach.

**Results:**

The data from this study suggest that a determining factor of a woman’s experience of pain during labour is the meaning she ascribes to it. When women interpret the pain as productive and purposeful, it is associated with positive cognitions and emotions, and they are more likely to feel they can cope. Alternatively, when women interpret the pain as threatening, it is associated with negative cognitions and emotions and they tend to feel they need help from external methods of pain control. The social environment seems particularly important in shaping a woman’s pain experience by influencing her interpretation of the context of the pain, and in doing so can change its meaning. The context and social environment are dynamic and can also change throughout labour.

**Conclusion:**

A determining factor in a woman’s experience of pain during labour is its perceived meaning which can then influence how the woman responds to the pain. The meaning of the pain is shaped by the social environment and other contextual factors within which it is experienced. Focussed promotion of labour pain as a productive and purposeful pain and efforts to empower women to utilise their inner capacity to cope, as well as careful attention to women’s cognitions and the social environment around them may improve women’s experiences of labour pain and decrease their need for pain interventions.

**Electronic supplementary material:**

The online version of this article (doi:10.1186/s12884-017-1343-3) contains supplementary material, which is available to authorized users.

## Background

The majority of women experience pain during labour and childbirth. For many women it is the most significant pain they will experience in their life. However, despite it being associated with the same fundamental physiological process, not all women experience labour pain in the same way. Women’s evaluations of labour pain can range from excruciating to pleasurable in different individuals or on different occasions [1, 2]. Some women manage the pain well, requiring minimal assistance and reporting positive experiences, whilst others do not cope well and request intervention in order to avoid or alleviate the pain [3]. Curiously, women have reported labour pain as a paradoxical experience of pain – one that is both excruciating but also desirable because of its positive outcome of the birth of their child [2]. It is thus clear that labour pain is a complex and unique experience of pain and, consequently, is challenging to manage.

As a result of the emerging complexity of the phenomenon, the current methods of supporting women through this experience may not be adequate. While a range of pain management strategies are available, pharmacological interventions are frequently used. Seventy-seven percent of women giving birth in Australia use pharmacological intervention for pain relief during labour, including regional analgesics (33%) and systemic opioids (20%) [4]. While epidural analgesics are recognised to be effective in managing pain, paradoxically they are not associated with more positive labour experiences in women [5, 6] and can contribute to reducing the rates of normal birth [4, 7–9]. Some women are so fearful of labour pain that they elect a caesarean section in order to avoid labour and vaginal birth altogether [10], and the fear itself can lead women who do labour to experience the pain as more intense and to report a more negative experience [11]. Compared to non-pharmacological methods of pain management, pharmacological methods are also associated with poorer outcomes for babies, including a higher rate of instrumental births and admission to special care, and decreased duration of breastfeeding beyond 6 weeks [12]. Overall, it is clear that current approaches to supporting women to manage labour pain do not always promote physiological birth, can diminish women’s experiences of labour and birth, and can have adverse effects on their babies’ health. In order to improve the support given to women during labour and birth we must first improve our understanding of labour pain and why women experience it so differently.

Modern pain science recognises that pain is a personal, subjective experience that is strongly linked to the social environment [13, 14]. Physical and emotional pain overlap both physiologically (based on the neural correlates of these experiences) and functionally (one can predict the other) [15], indicating that pain should be more comprehensively thought of as a driver to avoid physical as well as social threats to one’s wellbeing [16]. Pain is highly influenced by cognitive processes and is ultimately experienced within the context of its meaning to the individual [13, 14, 17, 18].

We can use this modern view of pain to re-examine our understanding of labour pain and why women experience it so differently. Labour pain literature has shown a correlation between cognitive processes and the experience of labour pain. Women who catastrophise pain [19, 20], have lower prior self-efficacy for labour [21] and have higher anxiety sensitivity ratings [22, 23] tend to experience more intense pain and take longer to recover postpartum [19–23]. Conversely, having a focussed and accepting state of mind as well as a known and trusted caregiver is associated with more positive pain experiences and decreased use of analgesics [3, 24, 25]. Interestingly, a woman’s attachment pattern prior to labour can also predict her experience of pain where more anxious attachment patterns are associated with a perception of pain as more threatening [26]. When viewed in the light of current understandings of pain these findings suggest that a woman’s perception of pain during labour is determined by a complex mix of psychosocial factors, in combination with what is happening in her body.

A recent Australian randomised controlled trial found positive effects of a birth preparation course incorporating complementary medicine techniques on reducing epidural use [27]. The course included acupressure, visualisation, breathing, massage, yoga and facilitated partner support. Likewise, a meta-analysis of non-pharmacological approaches for pain during labour also found reduced use of epidural as well as improved satisfaction with labour and birth [28]. This body of evidence demonstrates that non-pharmacological interventions that are focussed on changing the labouring woman’s thoughts, emotions and social environment can reduce her need for analgesia. What is unclear to date, however, is what effect these interventions are having on her experience of pain during labour and therefore what the possible mechanisms of effect are.

The overall aim of our body of work is to examine labour pain from the woman’s perspective so that we may develop a more complex and complete understanding of this phenomenon. This can then guide interventions to enhance women’s experiences of labour and childbirth, and reduce their need for pharmacological or surgical intervention. In 2014 we published the findings from the first part of our research [3]. This was one of the first studies to examine and describe the cognitive processes that occur in a woman’s mind during labour and how they relate to her experience of pain (based on her recall of the experience). Emergent from the data was the idea that a woman’s state of mind during labour sets the stage for the cognitive and evaluative processes that construct and give meaning to her pain experience. Two states of mind were identified – ‘mindful acceptance’ and ‘distracted and distraught’ – each having a different effect on women’s perception of pain, and women reported moving between the two states over the duration of labour. The present study aims to build on these findings. Taking into account the literature that suggests a strong influence by cognitive evaluative processes as well as the social environment, we aimed to further examine the link between these factors and the pain experience in labouring women.

## Methods

Phenomenology is a philosophy as well as a theoretical framework. It is based on the understanding that certain phenomena can only truly be understood from the perspective of the person experiencing them. This is appropriate when investigating complex subjective phenomena such as labour pain that can only be accessed through the conscious mind of the person experiencing it [16]. Furthermore, pain is a multidimensional construct that is perceived within a personal context. Phenomenology aims to capture lived examples of the phenomenon of interest within the context of the lives of the individuals experiencing it [29]. Interpretative Phenomenological Analysis (IPA) is a method of examining those lived examples and takes into account the fact that the researcher will play an active role in the process [29]. Phenomenology, using an IPA approach, can result in a deeper understanding of how and why a phenomenon exists [30].

### Participants

The women participating in this study were recruited through two large maternity services in Melbourne, Australia. Recruitment took place in the hospitals’ antenatal settings while women waited for their appointments. This included antenatal and fetal monitoring clinics at the hospitals, as well as community antenatal clinics associated with the hospitals. Nulliparous women in late pregnancy (>30 weeks gestation) who were not booked for a planned caesarean section and who were expecting a normal vaginal birth at the time of recruitment were invited to participate. Stratified purposive sampling was used in order to represent women at both higher and lower risk of complications, as well as women in different models of care: standard hospital care (midwifery-led care with no continuity), team midwifery care (some continuity of care) and caseload midwifery care (continuous care from one known midwife plus a backup midwife).

### Procedure

Women participated in two interviews, as well as completed three questionnaires during the study. The semi-structured pre- and post-birth interviews were conducted with researcher LW between December 2013 and January 2015. The pre-birth interview was designed to explore women’s thoughts and expectations about labour pain, and how they anticipated they would cope. This interview also allowed for the development of rapport between the women and the interviewer prior to the experience of birth. The post-birth interview [see Additional file 1] was designed to capture women’s experiences of labour pain and forms the focus of this paper. Women were asked to reflect on the labour and describe their experience from the onset of first stage of labour through to the birth of their baby. Prompts were given where necessary to encourage women to explore and describe their pain experience from sensory, affective and cognitive perspectives. Interviews were conducted in women’s homes within 3 weeks of giving birth and lasted between 45 and 90 min. One participant was unable to complete the post-birth interview so instead provided a written account of her experience. The questionnaire that was given to women after their post-birth interview included a section in which they could write additional comments about their labour and birth experience. Written comments in this section that related to their experience of labour pain were also included in data analysis. Women also consented to the collection of data relating to their pregnancy, labour and birth from their hospital medical records.

### Analysis

Interviews were transcribed verbatim and imported, along with the text from the open-ended question in the questionnaire and the written account, into NVIVO 10 software [31] for data management. Participant numbers are used in this paper and any names used within quotes have been changed to ensure anonymity. Data analysis was conducted according to the principles of IPA. Transcripts and written accounts were initially read to get a sense of the whole experience. Meaning units were then identified and organised into categories. Related categories allowed for the emergence of the themes of the text and finally allowed for a meaningful description of the investigated phenomenon. Coding was performed by LW and LJ, and checked by MD and RS. Any discrepancies in interpretation were discussed until agreement was reached.

## Results

### Participants

Twenty-one women aged 21–36 years participated in the study. Participant characteristics are presented in Table 1. Other important descriptors of women’s pregnancies and birth are presented in superscript after participant numbers at the end of quotes and are described in Table 2. This includes pregnancy risk levels, onset and progress of labour and mode of birth.

**Table 1:** Participant characteristics

	(*n* = 21)
Age, *M (SD)*	29.4 (3.5)
Level of education, *number*	
School less than year 12	2
Year 12 or vocational equivalent	5
Tertiary	13
Unknown	1
Onset of labour, *number*	
Spontaneous	5
Augmented	4
Induced	12
Birth outcomes, *number*	
Normal vaginal birth (unassisted)	10
Instrumental vaginal birth (vacuum/forceps)	4
Unplanned caesarean section	7
Pregnancy risk level, *number*	
High	8
Low	13

**Table 2:** Participant descriptors

Pregnancy risk level	Onset of labour	Mode of delivery
H = High	SPON = Spontaneous	NVB = Normal vaginal birth
L = Low	AUG = Augmented	IVB = Instrumental vaginal birth (vacuum/forceps)
	IND = Induced	CS = Caesarean section

### Overview of findings

Women’s descriptions of labour pain in this study demonstrate how complex this experience is. The overall theme to emerge from the data was that the pain a woman may be feeling during labour is given a meaning and it is the meaning of the pain that shapes the pain experience that she has and her ongoing response to it. Two major findings within this theme were made. First, the context of the woman’s pain experience shapes the emotional and cognitive values that she places on it to give it meaning. Second, this evaluative process is influenced by the social environment. Both the context and the social environment are dynamic and may change throughout her labour. The ultimate meaning of the pain determines whether the woman’s experience of pain is positive or negative, and importantly, whether she feels she can manage the pain without the need for external sources of pain control.

### The meaning of the pain

Women’s descriptions of pain were expressed with an emotional and a cognitive value that gave the pain personal meaning to the woman at that particular moment in her life. When emotions and cognitive evaluations were positive, the meaning of the pain was that it was productive and purposeful, and women felt they were able to self-manage the pain. Conversely, when emotions and cognitive evaluations were negative the meaning of the pain was that it was unnecessary or threatening and women would look for external sources, such as an epidural, to manage the pain. The context of the pain prompted this evaluative process.

#### Meaning: *The pain is productive and purposeful*. Women’s response: *I can cope*

In this scenario, women described the context of the pain as being associated with a desirable outcome – the birth of their child. Consequently, the meaning of the pain was that it was purposeful because it was working towards this goal.

…at the end of it you’re going to have a baby and you’ve been waiting your 9 months for this to happen and it’s the natural thing that’s going to happen, everyone goes through it. It’s not like you know you’ve been in a car crash and you’ve broken bones and stuff like that. This is something that’s completely natural. (2108)^H, IND, CS^

Oh basically I would just try and think of the end result and what was going to happen. I wasn’t actually thinking about the pain that I was in at the time. I just thought ‘this is happening for a reason’. (2104)^L, IND, CS^

Mentally you know it’s for a good reason. (1207)^L, AUG, NVB^

The cognitive value of the pain was positive. Women reasoned that the pain, particularly its intensity, was useful as it indicated the progress of their labours.

She was rocking me and, like, making me rock, which made it more intense but I knew that that was a good thing … you had it in your mind the whole time that the contractions were good even though they were painful, it was good because it was sort of tracking your progression and if anything if they got sort of closer together or more intense … well it doesn’t make you worried like if … if it was any kind of pain and you don’t know what it is or something I suppose you’d feel stressed about the pain. I never felt stressed about the pain or you know worried that my body … that something was wrong ever. (2106)^L, SPON, NVB^

… every contraction brings you closer to the goal, so you know it had to be done in order to give birth. So you’re not worrying that much, you’re just worrying about the intensity maybe. But, again, you know it’s intense. But it brings you to the goal. (2101)^L, AUG, NVB^

The emotional value of the pain was positive. Women experienced positive feelings in relation to the pain.

It was so tiring and exhausting but so rewarding. (1205)^L, SPON, IVB^

…and then probably an hour after my waters broke I started getting the contractions. But again I wasn’t scared because I knew what was coming, I was like, oh yeah, so this is a good step, this is a good step even though it was painful. (2103) ^L, IND, IVB^

When women interpreted the pain as being productive and purposeful, their response to the pain was that they could cope. They did not seek external methods of pain control – they felt that they possessed the inner strength to manage the pain.

I would say it’s painful but it’s manageable. (2103)^L, IND, IVB^

You can cope with it, you can definitely cope with it. The body will find a way. (2101)^L, AUG, NVB^

Pain was all worth it and it is hard but not impossible. It hurt but it wasn’t impossible pain … the pain is bearable, you know you can get through it. (2111)^L, IND, NVB^

#### Meaning: *The pain is threatening*. Women’s response: *I need help*

In this scenario, the context of the pain was that it was not productive. Women’s interpretation of the situation, often shaped by a sense that either their progression, or the intensity of the pain, did not match their expectations, lead them to experience the pain as not working towards a goal. Thus, the meaning of the pain was that it was a threat to her physical or emotional wellbeing and it urged her to call for help.

The pain was getting worse and all I kept on saying to Peter was ‘I want the epidural … if this is the pain I’m having at 3cm what is it going to be at 8cm?’ (2201)^L, SPON, NVB^

I was like okay, hopefully it’s 10cm, I was convinced I must have been and then they checked and I was only six and then it was the most sinking feeling that I’ve ever experienced, and then that was it, I was like I’m done, time for the epidural … I’d gone through all that pain for nothing … (2107)^H, IND, CS^

The cognitive value of the pain in that context was negative. Women’s thoughts about the pain suggested that the pain either wasn’t perceived as productive, or there was a mismatch between their expectations and their experience. As a result women could not embrace the pain or work with it.

I basically felt like my insides were turning inside out. It just … it felt like my stomach was going to fall out. The only thing is I just didn’t have that urge to push, I think if I had that urge to push and if I was dilated that little bit more it probably would have been that little bit easier for me but because I didn’t have that sensation … I couldn’t do anything with it, it was just a massive massive stomach ache. (2104) ^L, IND, CS^

Once I realised that I was only 4cm dilated as well like that’s pretty disheartening and they got the doctor in as well and said that they’d need to induce me. And so I asked for an epidural because I couldn’t handle the idea of it being more intense and more pain and possibly much, much longer. I’d given up. (2106)^L, SPON, NVB^

… and they’d said, well, you must be ten now ‘cause you’re trying to push and we should check and I was still 6cm, so that was my give up point and that’s when I went for morphine and the epidural … So by that point I think I’d just given up and I don’t know if the pain became a little bit irrelevant, it still hurt because I’d kind of handed over all the control to the drugs, in a way. (2107)^H, IND, CS^

The emotional value of the pain in that context was also negative. It elicited negative emotions that coloured the whole labour experience in a negative way.

It was the worst thing in the whole entire world and I never want to go through it again. (2114)^H, IND, IVB^

And then the pain just got … oh it was horrendous, it was horrible and I think just because it was so quick and I was already tired from it, like just being tired from being awake the whole time was horrible. (2204)^H, IND, NVB^

Maybe more expecting the twisting and the knotting and perhaps a little pressure. But just the absolute sharpness of the [pain]. Really scary. (2203)^H, SPON, NVB^

### The social environment

Aspects of the social environment appeared to be highly influential in shaping women’s perception of pain. The social environment, which included caregivers, support people, hospital staff and even strangers, was able to influence the woman’s state of mind and therefore the cognitive and emotional values of her pain. In addition, the words or actions of the people around her were able to change the context of the pain. In doing so, these individuals could shape the meaning that the woman gave to the pain i.e. whether she perceived the pain to be productive and purposeful and that she could cope, or whether it was threatening pain and she felt she needed help from external sources of pain control.

#### Social environment: *I am in pain but I feel safe. I can cope*

When the people around the labouring woman were known, trusted and calm, she had a sense of being safe. The woman felt emotionally supported which may have helped the pain feel less threatening.

Having him there didn’t alleviate the pain, it just was comforting … he was like my life net … I don’t know how women would do it alone. It would be awful. (2107)^H, IND, CS^

In particular, when the caregivers created a calming atmosphere, it kept the woman’s mind in a calm state and helped her avoid pain catastrophising.

I think everyone was just really calm around me like my midwife was very calm. Every time she spoke to me she was very soothing and ... there was no panic in her voice … I think I would have panicked if she was more panicky. (2202)^L, AUG, CS^

Her state of mind could be further influenced by encouragement and instruction from her caregivers. This could help her stay focussed.

Yeah, definitely people talking me through it you know you’ve got to slow down your breathing, breathe in through your nose, breathe out through your mouth, just talking you through it was very helpful, to help you re-centre and get back on track. (2106)^L, SPON, NVB^

Caregivers’ words were able to change the context of the pain and thereby influence its meaning to the woman (see quote 2201). The words of her support people were also able to shift her self-belief in her capacity to self-manage the pain.

I just kept saying that I couldn’t do it. And then Sally was like “no, you can, you can do it, you can keep doing it, you’ve been doing it, come on”, you know? And that was good, that was good to have her, she was perfect at that time. (2111)^L, IND, NVB^

Overall, when the woman was made to feel safe through the social environment that she was in, she felt more capable to tune into her body, be accepting of the experience and ‘go with the flow’.

You don’t care what’s happening around you as long as you know they’ll take care of you and you’ll be in good hands … and then you can just let go, you just do what you have to do and just go with the flow. (2101)^L, AUG, NVB^

#### Social environment: *I am in pain and I feel unsupported. I need help*

When a woman lacked the support or presence of her preferred caregiver or support person it had the capacity to influence her pain experience. It gave her a sense of being alone and emotionally unsafe.

You’re in this most incredible pain that I’ve … I wouldn’t even know what to compare it to and then they make your partner go home, which is just absurd because you need that support … you don’t want to do it alone, it’s horrible. (2107)^H, IND, CS^

The presence of strangers or others, whom the woman did not want present, could interfere with her focus. This distraction was unhelpful and even emotionally intrusive during her pain experience.

Because I know they really can’t help me out and it’s ... it’s in fact distracting and it stresses me out. I don’t want to see anybody ‘til I have my baby because I’m really in that much pain, I don’t want to see anybody at all. Your husband is the person that you can share ... but not with everyone … (2102)^L, IND, CS^

The context of the pain could be influenced by what a woman was told by her caregivers, even if it was simply reporting as assessment finding. In this way, caregivers had a powerful influence over the woman’s pain experience because they had the capacity to change the context (from the woman’s perspective), and therefore the meaning of the pain.

When they told me I was 3cm … that’s probably the main thing out of my whole labour that really got me, I started crying ‘cause I was just so upset because like you hear you have to be this many centimetres … when you’re at hospital, and women can cope between these centimetres and … because I was only 3 … I was like not coping with it… But I reckon if they were to tell me that no, look, you are 8cm, this is the pain at 8cm, I would have been like alright, I’m managing with the gas then. (2201)^L, SPON, NVB^

The woman would read the social environment in order to understand the context of her pain. It was not just the words spoken by caregivers, but also the makeup of who was present, as well as their body language, which could subsequently lead her to feel concerned that something was wrong.

…having like a hundred people in the room and their faces all looked petrified. A hundred faces really stressed is pretty scary. (2114)^H, IND, IVB^

In different ways, these characteristics of the social environment could result in the woman experiencing labour pain as threatening and gave her a sense that she needed help.

## Discussion

### Summary of findings

The data from this study suggest that a determining factor of a woman’s experience of pain during labour is its meaning. When women interpret the pain as productive and purposeful, it is associated with positive cognitions and emotions, and they are more likely to feel they can cope. Alternatively, when women interpret the pain as threatening – to either their physical or emotional wellbeing – it is associated with negative cognitions and emotions. When this is the case they tend to feel they need help from external methods of pain control.

The context of the experience influences the meaning of the pain to the woman. Despite the fact that in all cases the process is the same, i.e. the woman is proceeding through labour, for different women this can have different meanings. For some, the pain experience associated with being in labour is acceptable and the pain is seen as necessary (i.e. purposeful). For others, being in labour and the associated experience of pain is not embraced and may be associated with fear or catastrophising thoughts (i.e. threatening). Throughout labour the context of the pain is dynamic and its meaning is changeable – a woman can associate the pain with progression of labour and a sign that she is nearing the birth, or it may be associated with apparent lack of progression, or with artificial progression, or even with progression towards a surgical birth. In each of these cases, the meaning of the pain then changes.

The social environment seems particularly important in shaping a woman’s experience of pain during labour. The people around her can influence her interpretation of the context of the pain, and in doing so can change its meaning. The presence of certain people can influence a woman’s sense of feeling safe or of feeling vulnerable, as well as the thoughts she has towards the pain. She may use the words, actions or expressions of those around her to help her understand the context and thereby construct the meaning of her pain. As the social environment changes throughout her labour, so too can her interpretation of the meaning of the pain. The pain can become more threatening or be seen as more productive, based on a woman’s reaction to variations to the social context she is labouring in.

Ultimately, it is the meaning of the pain that matters to the woman and will influence her ability to cope. An intense pain that is purposeful and that the woman associates with her labour progressing (i.e. it’s productive) is very different to an intense pain that the woman is interpreting as a threat to her baby or herself.

### Interpretation in light of the literature

The individual meaning of a pain experience is a dimension of pain that has not been greatly explored in the existing pain literature, and yet it is an implicitly accepted dimension. The placebo effect – a phenomenon that is routinely controlled for in much clinical research – is a striking example of how context can change a person’s pain experience [32]. Moseley and Arntz [17] demonstrated experimentally that a change in the context of a noxious stimulus resulted in a change in the perceived intensity and unpleasantness of the subsequent pain experience. This correlates with the findings from the present study where the context of a woman’s labour experience reflects the perceived intensity and the qualitative characteristics of pain. Our previous work demonstrated that women describe their labour pain experience using either positive, negative or ambivalent terms [3] suggesting that not all labour pain feels the same. This relates to Lundgren & Dahlberg’s [2] findings that labour pain can be a contradictory experience, i.e. both positive and negative, because its context is a desirable one (giving birth to a child). The data from the present study have demonstrated the complexity of pain experiences due to the varied personal meaning of the pain to the individual. Labour pain is a unique experience where a woman can derive a positive meaning of the pain that enhances her capacity to cope.

The data in this study suggest that the people around the labouring woman can shape her pain experience. It is well known that the continuous presence of a known caregiver can improve labour and birth outcomes for women, including a reduction in the use of analgesics [25]. A review of qualitative literature examining women’s experiences of coping with pain during childbirth reported that support from known, trusted caregivers to whom a labouring woman feels emotionally connected makes her feel safe and enhances her ability to cope with pain during labour [33]. The data from the present study may help us understand how this effect is taking place. The data demonstrate that caregivers can influence the woman’s pain experience by influencing her cognitions and emotions towards the pain and, in doing so, can change the meaning of the pain to the woman. This subsequently affects her sense of her ability to cope. By facilitating a state of focus and calm, as well as cultivating positive cognitions about the pain, caregivers can tacitly reassure her that the pain is productive and purposeful. This state of mind is akin to a ‘mindful acceptance’ state that has previously been demonstrated to allow the labouring woman to accept the pain as non-threatening and to work with it [3]. Conversely, a ‘distracted and distraught’ state of mind is associated with pain catastrophising and a sense of helplessness. The data from the present study show that when caregivers explicitly or inadvertently suggest to the woman that something is wrong or that she is not progressing at the expected rate, it changes her cognitions and emotions towards the pain and leads to a state of mind with similar qualities to a ‘distracted and distraught’ state. This suggests that the social environment may be a key regulator of a woman’s state of mind and, in doing so, can change her pain experience.

The data in this study also demonstrate that women can derive a sense of safety or of vulnerability from the people around them during labour and that this seems to be linked to their interpretation of the pain as productive and purposeful, or threatening. Pain science now recognises pain to be the output of a threat-response system [34] that has been activated by implicit or explicit threats to one’s safety [35]. Pain is a homeostatic emotion that motivates the individual to do something about it – specifically, actions that are associated with enhanced chances of survival and thus a sense of safety [36, 37]. The data from this study suggest that the pain a woman feels during labour will be influenced by her sense of safety, or of vulnerability, influenced by the social environment. If the people and the interactions going on around her trigger a sense of vulnerability, it makes evolutionary sense that a woman’s pain experience will change to a more threatening feeling that will then motivate further safety-seeking behaviours.

Notably, one purpose of labour pain may be as a trigger to elicit social support in a time of urgent need [38]. The response to the woman’s call for help is facilitated by the experience of empathy for her pain in the caregiver. A meta-analysis by Lamm et al. [39] has revealed that the empathy networks in the brain overlap with the areas of the brain seen to be involved in a pain experience. Social connections between the labouring woman and others may serve her an important purpose in eliciting a response in her caregivers through their deep inner understanding of her pain. Through the empathic response she receives, her pain experience is validated and she is emotionally and cognitively supported to understand, accept and cope with her pain. Further, Eisenberger [15] demonstrated that the neurophysiology of physical and emotional pain overlap and that emotional pain can heighten physical pain (and vice versa). In this regard, it may be that the social pain of being alone or feeling vulnerable during labour contributes to the woman’s physical pain experience.

The findings of this study identified a further value of labour pain: some women use their pain during labour to self-monitor their progression. Changes in the temporal properties such as an increase in frequency of contractions, and/or an increase in intensity, can signal to women that they are getting closer to the birth and thus influence their interpretation of the pain to mean that it is productive. This is an empowering feeling to have and may contribute to a sense of control that many women seek during labour [40]. Caregivers should also be aware of the potential to negatively influence the pain experience by reporting a woman’s degree of cervical dilation. The data in this study suggest that being told about the degree of dilation can suddenly shift a woman’s state of mind from one of coping with the pain to not being able to cope with the pain and requesting analgesia. The woman may interpret that measurement to mean that she is not progressing at the desired rate and therefore her pain is not productive or if she is already feeling challenged by the pain, it may lead her to predict that she will not have the capacity to cope as labour progresses. If in fact the woman is experiencing a slow progression of labour, caregivers may need to take care to ensure the reports of cervical dilation are accompanied by strategies to help the woman remain confident and promote the progress of her labour, to help prevent her experiencing pain as threatening.

Finally, we would like to acknowledge that while it is now clear that each woman’s unique cognitions, as well as her social environment during labour, are key determinants of her pain experience, she must also work with her body. There are some women who may have long, challenging and exhausting labours for various anatomical and physiological reasons. These women may have ‘ideal’ cognitions and social support to have a positive experience of labour, however the physical contribution to their labour pain makes the process difficult and their pain may become unmanageable. It is here that known, trusted caregivers who are tuned into her cognitively and emotionally, may be better able to offer the right type of support at the right moment.

We hope that the findings of this study inform the development and refinement of interventions to support women and their pain experience during labour. The results of this study suggest that interventions that encourage positive cognitions and emotions about labour pain, and promote labour pain as a productive and purposeful pain, may improve women’s pain experience and, importantly, her capacity to cope. The social environment is a key influencer and the findings of this study may be useful to all carers and support people of labouring women.

### Strengths and limitations

We believe that this study investigates important and under-researched concepts relating to labour pain. We have chosen a research strategy that enables us to seek a more complete understanding of the experiential phenomenon of labour pain and have interpreted the findings in light of modern pain science. Through this more sophisticated understanding of labour pain, better strategies to support women during labour and birth may be developed.

The findings of this study should be interpreted taking into account that the demographic factors of participants are not representative of all women giving birth. Despite women being recruited from two different maternity hospitals in various models of care, as well as differing pregnancy risk levels, this study focussed only on nulliparous women’s experiences. Over half of these women had completed tertiary level education.

In addition, the interview relied on recall up to 3 weeks after women’s labours. Previous work, however, has demonstrated that women’s recall of their labour experiences is surprisingly accurate even years after the event (see Niven & Murphy-Black [41] for review).

Finally, because of the limited research that has focussed on the cognitive processes that shape a woman’s pain experience during labour, it is important that these findings be used to as foundation for further research.

## Conclusion

This investigation into labour pain has helped to deepen our understanding of this unique and complex experience. A determining factor in a woman’s experience of pain during labour is its perceived meaning. The meaning influences how the woman responds to the pain – either productive and purposeful pain that she feels she can cope with, or threatening pain that she feels she needs help to alleviate. The meaning of the pain is shaped by the context within which it is experienced. The social environment plays a powerful role in influencing the woman’s cognitions and emotions, thereby helping construct the meaning of her pain.

Focussed promotion of labour pain as a productive and purposeful pain, and efforts to empower women to utilise their inner capacity to cope, as well as careful attention to women’s cognitions and providing a supportive social environment during labour, may improve women’s experiences of labour pain and decrease their need for pain interventions. In addition, these findings emphasise the importance of individualised care for each labouring woman as determined by her unique experience.

## Additional file

Additional file 1: Post-birth interview guide. (PDF 88 kb)

## Abbreviations

AUG: Augmented labour
CS: Caesarean section
H: High risk
IND: Induced labour
IPA: Interpretative Phenomenological Analysis
IVB: Instrumental vaginal birth (vacuum/forceps)
L: Low risk
LJ: Lester Jones
LW: Laura Whitburn
M: Mean
MD: Mary-Ann Davey
NVB: Normal vaginal birth
RS: Rhonda Small
SD: Standard deviation
SPON: Spontaneous onset of labour
