# Supplementary material for: The meaning of labour pain: how the social environment and other contextual factors shape women’s experiences
Source: BMC Pregnancy Childbirth. 2017 May 30;17:157. doi: 10.1186/s12884-017-1343-3 (PMC5450354; doi:10.1186/s12884-017-1343-3)
Supplement: Additional file 1: — Post-birth interview guide. (PDF 88 kb) [file 12884_2017_1343_MOESM1_ESM.pdf]

LA TROBE UNIVERSITY

Judith Lumley Centre
Faculty of Health Sciences

RESEARCH NUMBER

☐☐☐☐

Interview Guide – Post-birth

RESEARCHERS
Prof Rhonda Small (Judith Lumley Centre)
Dr Mary-Ann Davey (Judith Lumley Centre)
Mr Lester Jones (Department of Physiotherapy)
Ms Laura Whitburn (Judith Lumley Centre)

Post-birth interview questions/guide

Part 1 – Labour time-line

Based on the medical notes, or in discussion with participant, outline the labour by creating a time line.<br>• Note length of labour<br>• Add to timeline major events e.g. changes in location, changes in caregivers, interventions, other significant events<br>• Note method of delivery

Post-birth interview questions/guide

Part 2 – Pain during labour

• For each part of the labour (using events identified above) describe your labour pain experience
**PERIOD 1:**<br>Description of pain- thoughts/emotions/intensity<br><br>Meaning of the pain<br><br>Contextual influences<br><br>Social environment influences<br><br>Mindset - focussed/distracted<br><br>Interruptions?<br>To labour<br>To focus
**PERIOD 2:**<br>Description of pain- thoughts/emotions/intensity<br><br>Meaning of the pain<br><br>Contextual influences<br><br>Social environment influences<br><br>Mindset - focussed/distracted<br><br>Interruptions?<br>To labour<br>To focus

Post-birth interview questions/guide

**PERIOD 3:**<br>Description of pain- thoughts/emotions/intensity<br><br>Meaning of the pain<br><br>Contextual influences<br><br>Social environment influences<br><br>Mindset - focussed/distracted<br><br>Interruptions?<br>To labour<br>To focus
**PERIOD 4:**<br>Description of pain- thoughts/emotions/intensity<br><br>Meaning of the pain<br><br>Contextual influences<br><br>Social environment influences<br><br>Mindset - focussed/distracted<br><br>Interruptions?<br>To labour<br>To focus

Post-birth interview questions/guide

**PERIOD 5:**<br>Description of pain- thoughts/emotions/intensity<br><br><br>Meaning of the pain<br><br><br>Contextual influences<br><br><br>Social environment influences<br><br><br>Mindset - focussed/distracted<br><br><br>Interruptions?<br>To labour<br>To focus

Post-birth interview questions/guide

Part 2 – Overall impression of labour pain

What was your overall impression of labour pain? Compared to expectations?

Was it better/worse/as expected? What about it made it better/worse than expected?

What do you think influences labour pain? What makes it better/worse?

Effect of thoughts/mindset on labour pain?

Effect of people on labour pain?

Is there anything you wished was different about the labour pain?
